# Supplementary figures and images for: Distinct spermiogenic phenotypes underlie sperm elimination in the Segregation Distorter meiotic drive system
Source: PLoS Genet. 2021 Jul 6;17(7):e1009662. doi: 10.1371/journal.pgen.1009662 (PMC8284685; doi:10.1371/journal.pgen.1009662)

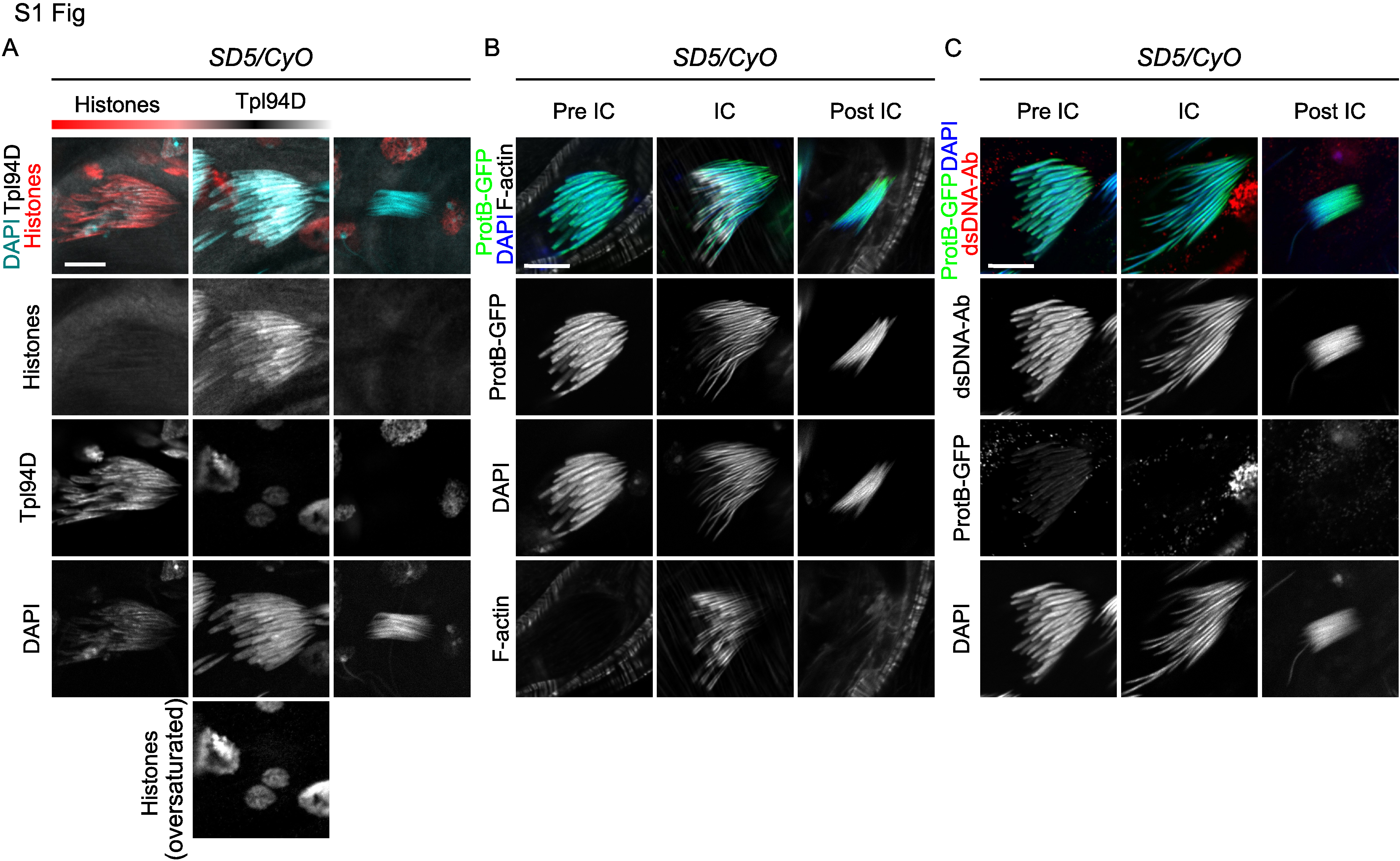

Supplement: S1 Fig — Confocal images of individual spermatid cysts from SD5/CyO; protB-GFP control testes stained in (A) with a pan-histone antibody (red), an antibody against the Tpl94D transition protein (white) and DAPI (cyan); in (B) with phalloidin (F-actin; white) and DAPI (blue) and in (C) with an anti-dsDNA antibody (dsDNA-Ab; red) and DAPI (blue). Scale bar: 10μm. (TIF) [file pgen.1009662.s001.tif]

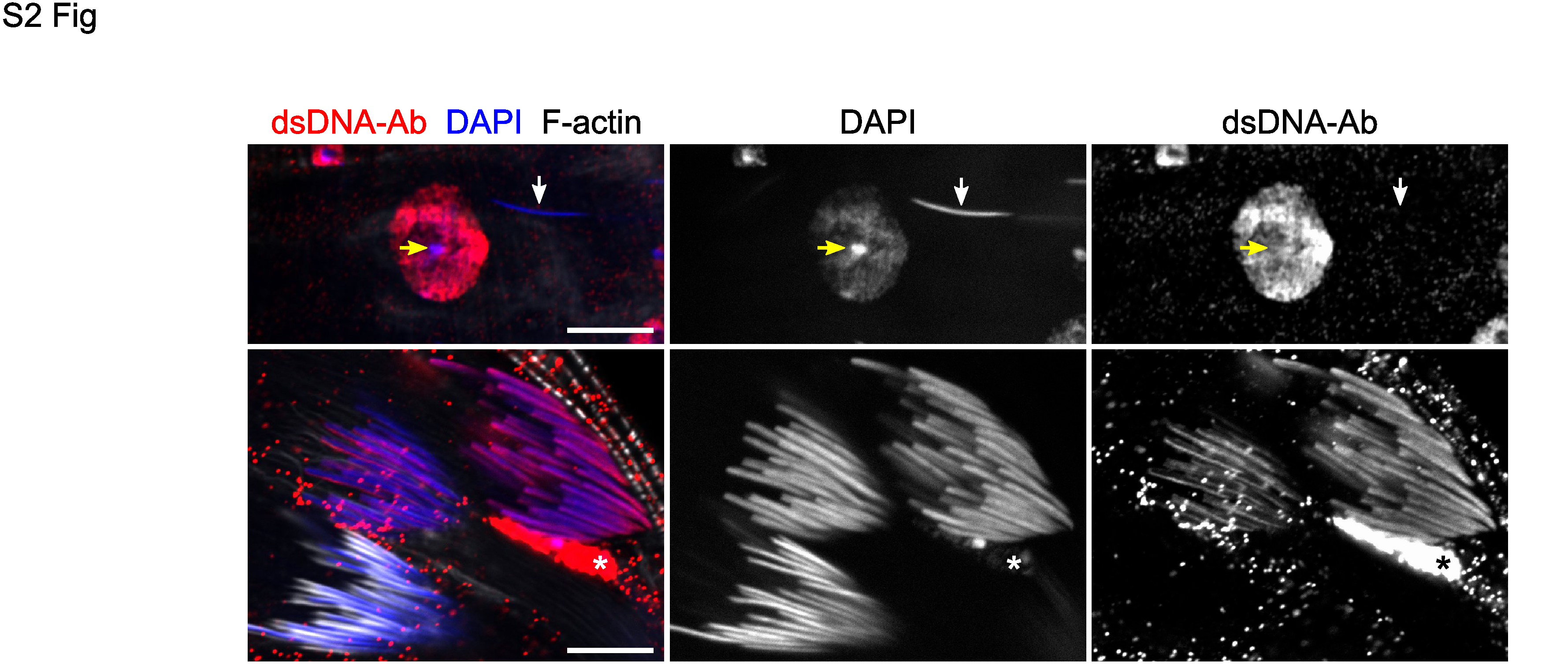

Supplement: S2 Fig — Confocal images of a whole-mount testis stained with DAPI (blue), phalloidin (F-actin; white) and an anti-dsDNA antibody (dsDNA-Ab; red). Top panels show the nucleus of a somatic cell next to a sperm cell nucleus (arrow). While the somatic nucleus is brightly stained with the anti-dsDNA antibody, the sperm nucleus is impermeant to it. DAPI staining intensity is proportional to DNA compaction and brightly stains highly compacted DNA such as heterochromatin (yellow arrow) in somatic nuclei. On the opposite, the anti-dsDNA staining is inversely proportional to chromatin compaction and is weak in heterochromatic regions. Bottom panels show three cysts of 64 spermatid nuclei at different stages. The two cysts on top contain elongating spermatid nuclei before individualization. These nuclei are stained with the anti-dsDNA antibody. The bottom cyst contains nuclei which have been invested by individualization actin cones. At this stage, nuclei are negative for the anti-dsDNA staining because of the high compaction of chromatin. Asterisk indicates a somatic nucleus. Scale bar: 10μm. (TIF) [file pgen.1009662.s002.tif]

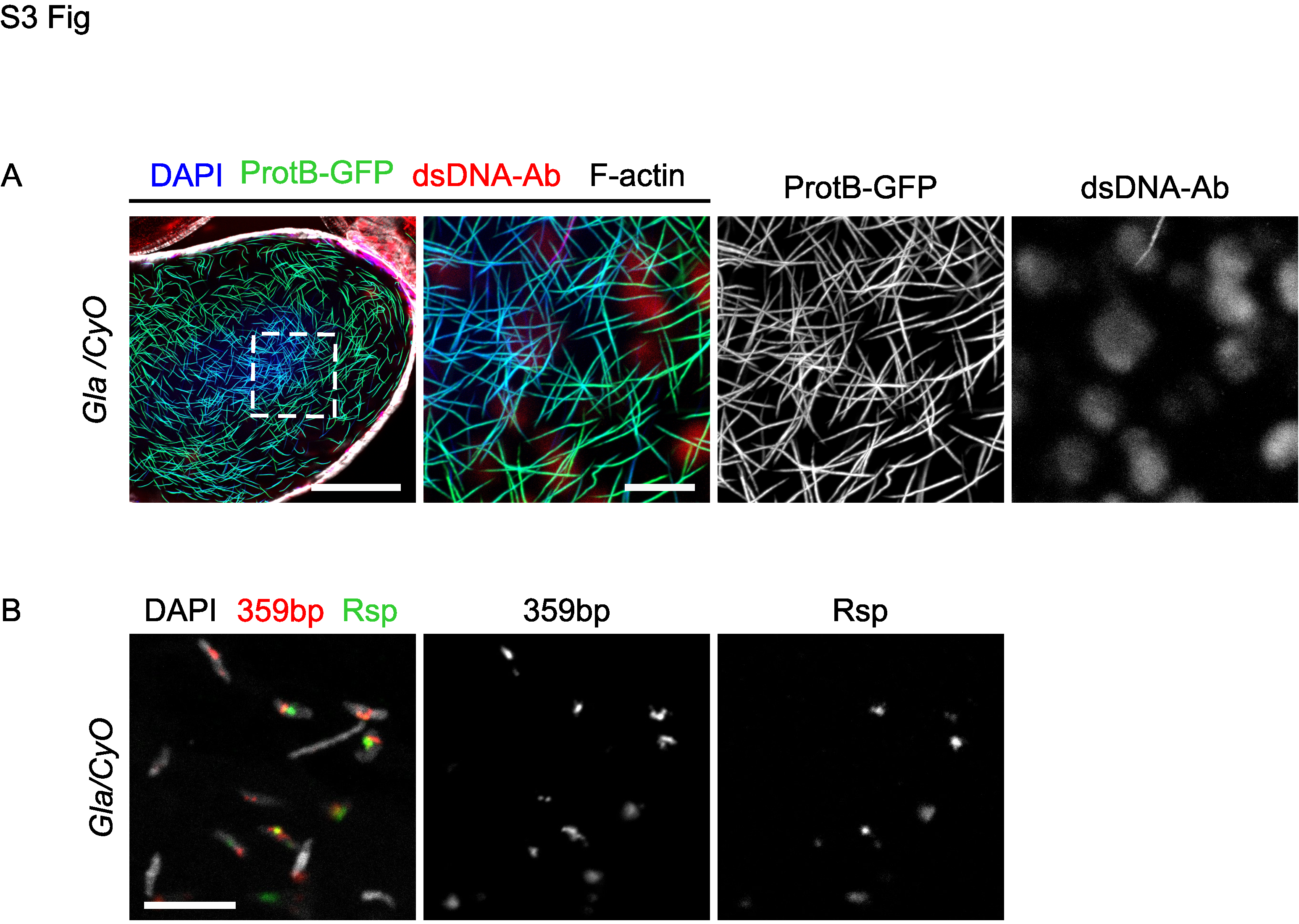

Supplement: S3 Fig — (A) Confocal images of a whole-mount seminal vesicle from a Gla/CyO; protB-GFP control male stained with an anti-dsDNA antibody (dsDNA-Ab; red), DAPI (blue) and phalloidin (F-actin; white). A wide view of the seminal vesicle is shown on the left panel (scale bar: 50μm). The dashed white square corresponds to a magnified region shown on right panels (scale bar: 10μm). Almost all sperm nuclei are negative for the anti-dsDNA antibody and are thus properly compacted. (B) DNA-FISH performed on seminal vesicle contents with a Rsp probe (green) and a probe for the 359 bp satDNA (red) on the X chromosome as a control (scale bar: 10μm). Sperm nuclei appear larger in all panels because they have been treated with DTT to facilitate probe penetration. (TIF) [file pgen.1009662.s003.tif]

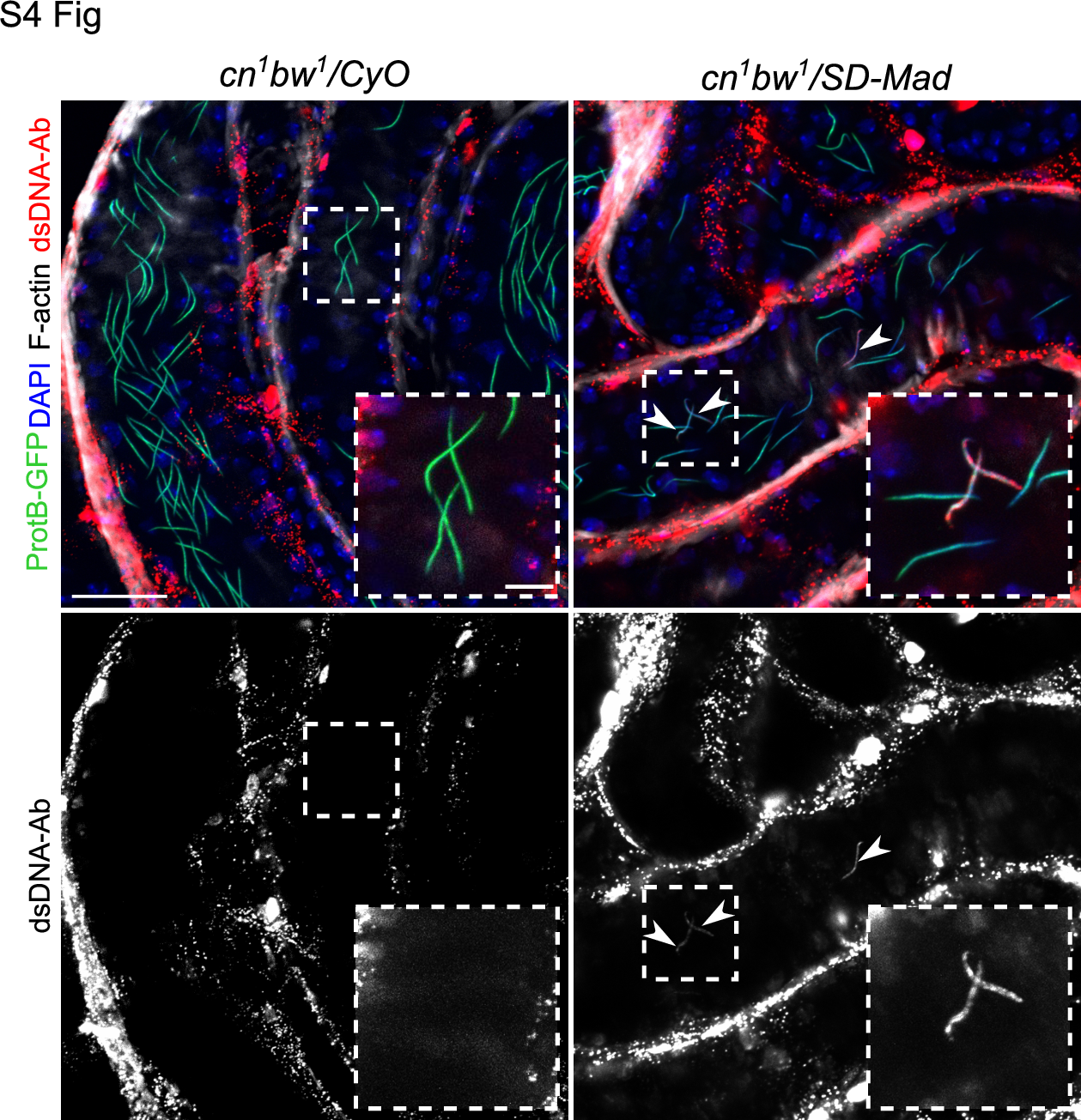

Supplement: S4 Fig — Confocal images of the seminal receptacle from a w1118 female mated to a cn1 bw1/CyO; protB-GFP control male (left) or a cn1 bw1/SD-Mad; protB-GFP male (right) and stained with DAPI (blue), an anti-dsDNA antibody (dsDNA-Ab; red) and phalloidin (F-actin; white). Right images show sperm nuclei stained with the anti-dsDNA antibody (arrowhead). Over ten seminal receptacles analyzed from females mated to cn1 bw1/SD-Mad; protB-GFP males, only one contained anti-dsDNA positive sperm nuclei (10/160). Scale bars: 20μm in full size images and 10μm in insets. (TIF) [file pgen.1009662.s004.tif]

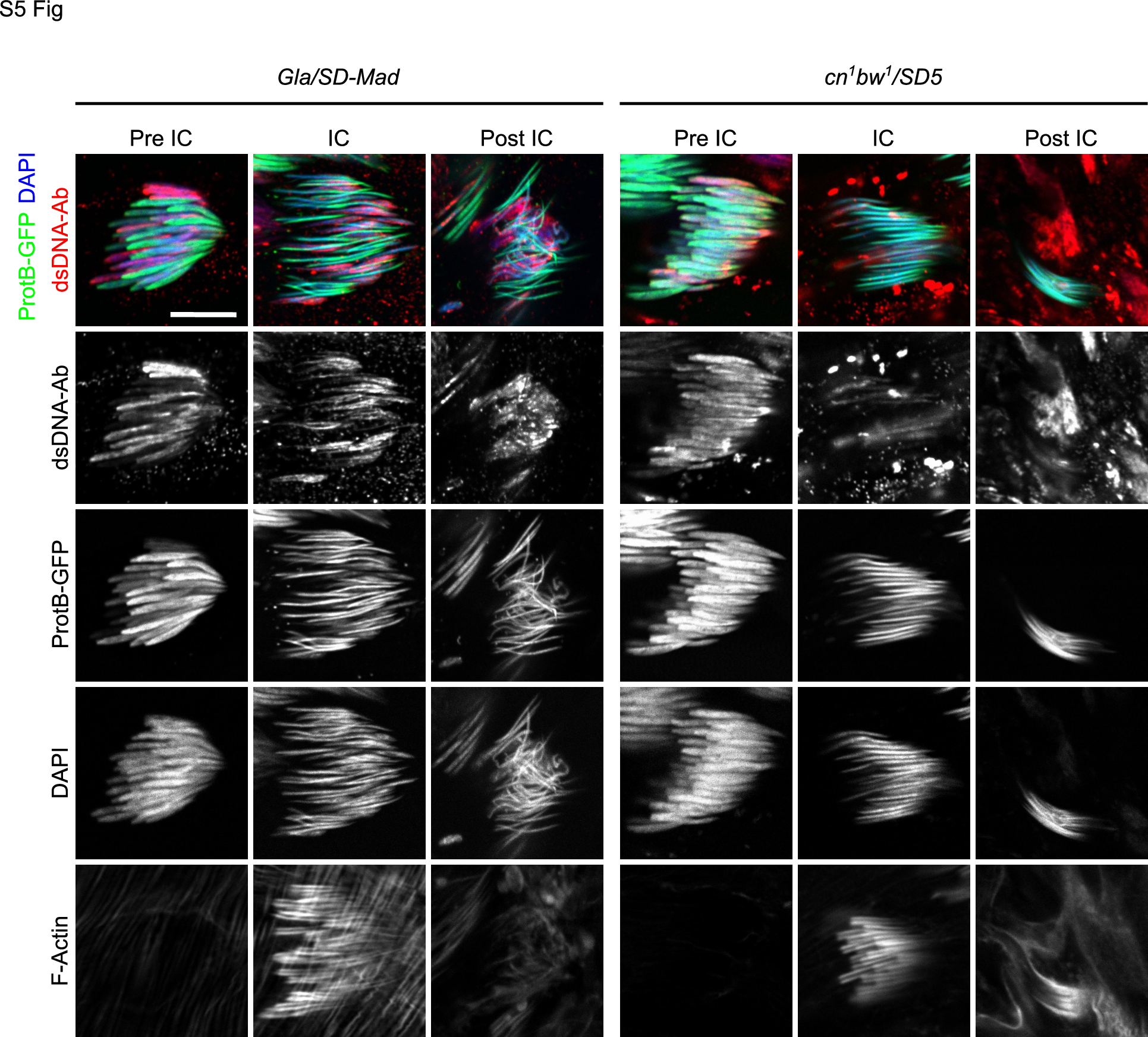

Supplement: S5 Fig — Confocal images of whole-mount testes from Gla/SD-Mad; protB-GFP and cn1 bw1/SD5; protB-GFP males stained with an anti-dsDNA antibody (dsDNA-Ab; red), DAPI (blue) and phalloidin (F-actin, white). In Gla/SD-Mad, many abnormally-shaped nuclei that are weakly stained with ProtB-GFP and brightly stained with the anti-dsDNA antibody are visible in pre-IC, IC and post-IC cysts. In cn1 bw1/SD5 testes, abnormally-shaped anti-dsDNA positive nuclei are less frequent. Bundles of post-IC spermatid contain needle-shaped anti-dsDNA positive nuclei. Scale bar: 10μm. (TIF) [file pgen.1009662.s005.tif]

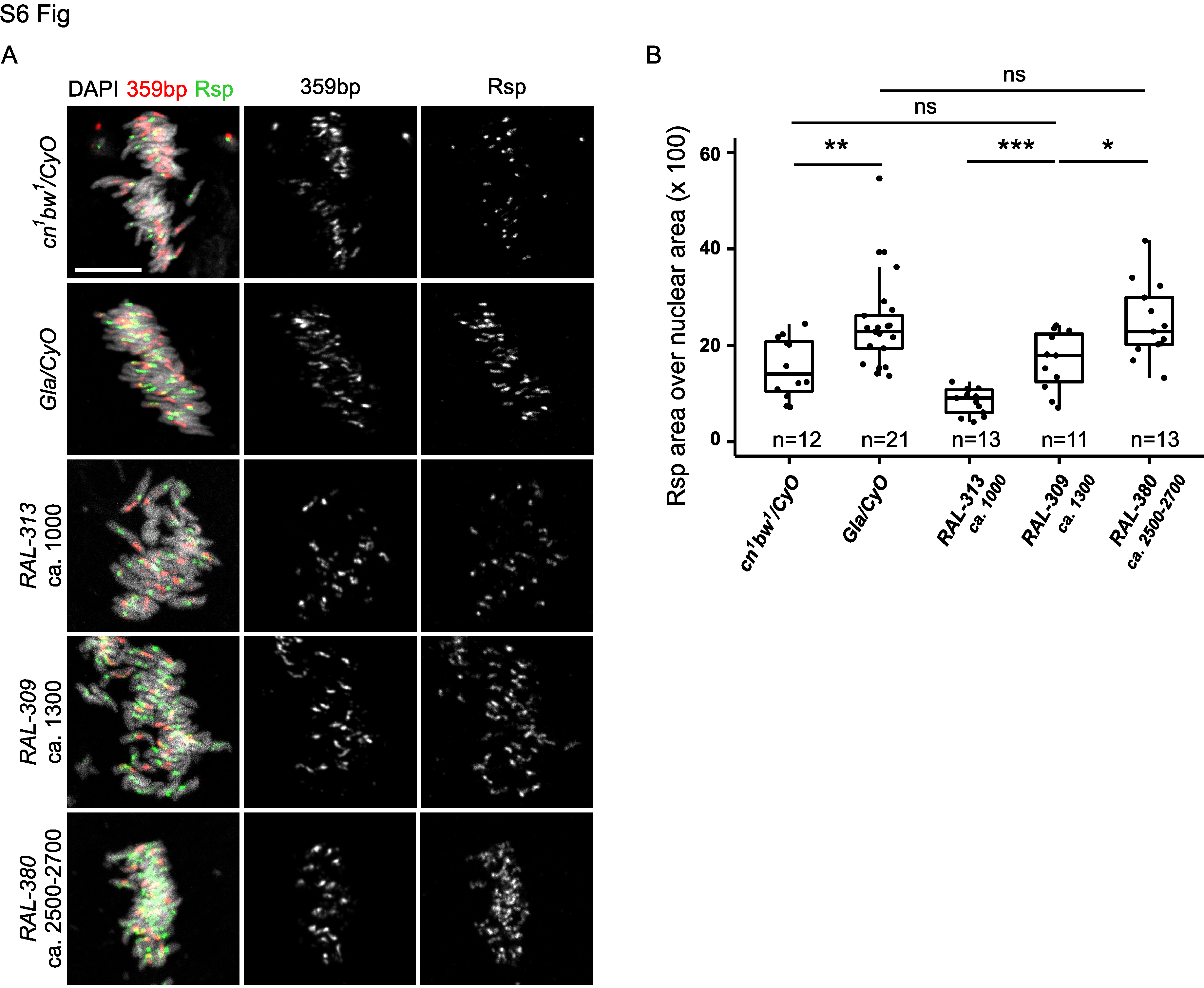

Supplement: S6 Fig — (A) DNA-FISH on squashed testes from cn1 bw1/CyO, Gla/CyO, RAL-313 and RAL-309 and RAL-380 males performed with a Rsp (green) and a 359 bp (red) probe. Scale bar: 10μm (B) Box plot showing the ratio of Rsp signal area over nuclear area expressed as a percentage. For each genotype, the number of isolated nuclei analyzed is indicated. The area of Rsp signals is larger in Gla spermatid nuclei compared to cn1 bw1 ones, in agreement with molecular quantification. Area of Rsp satDNA in RAL strains are also proportional to copy numbers determined by qPCR. Wilcoxon test, non-significant (ns) p-value>0.05, * p-value <0.05, ** p-value<0.01, ***p-value<0.001. (TIF) [file pgen.1009662.s006.tif]

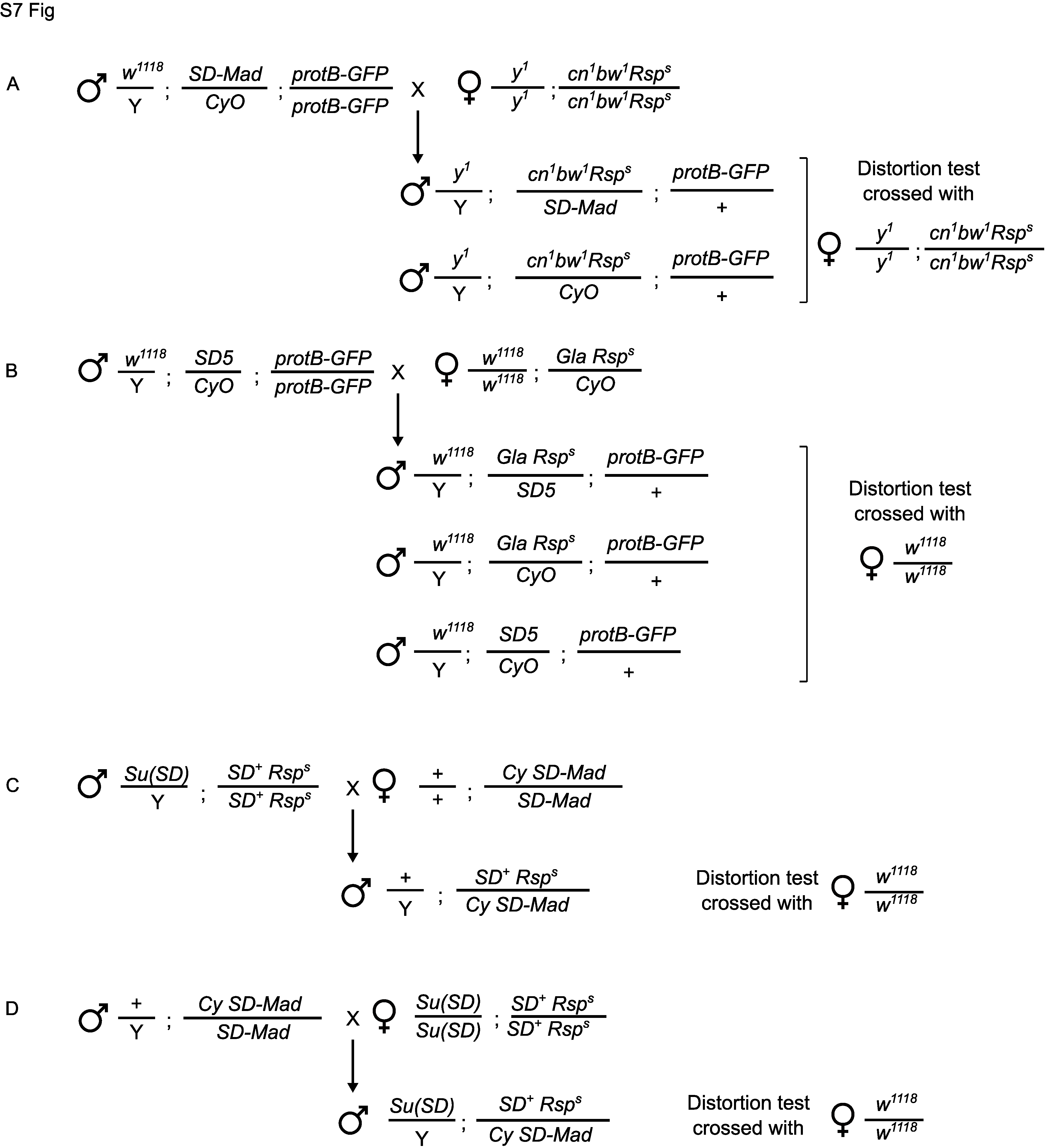

Supplement: S7 Fig — (TIF) [file pgen.1009662.s007.tif]

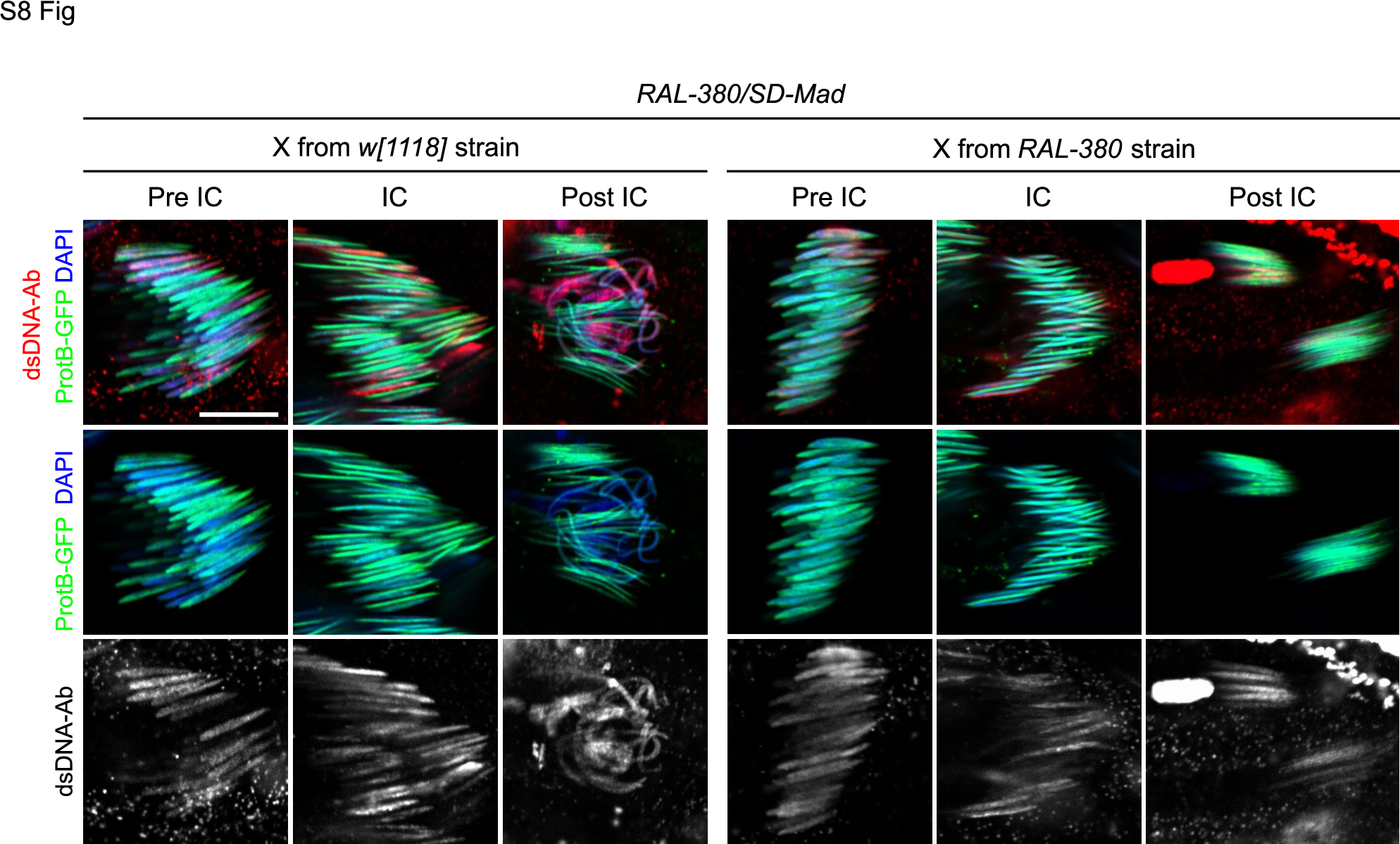

Supplement: S8 Fig — Confocal images of RAL-380/SD-Mad; protB-GFP testes carrying the X chromosome from the RAL-380 strain with Su(SD)X-380 suppressor (right panels) or the w1118 chromosome (left panels). Protamine incorporation and individualization appear less disturbed in presence of Su(SD)X-380. Bundles of individualized spermatid nuclei (post-IC) are less disturbed but include many needle-shaped nuclei positively stained with the anti-dsDNA antibody. Scale bar: 10μm. (TIF) [file pgen.1009662.s008.tif]

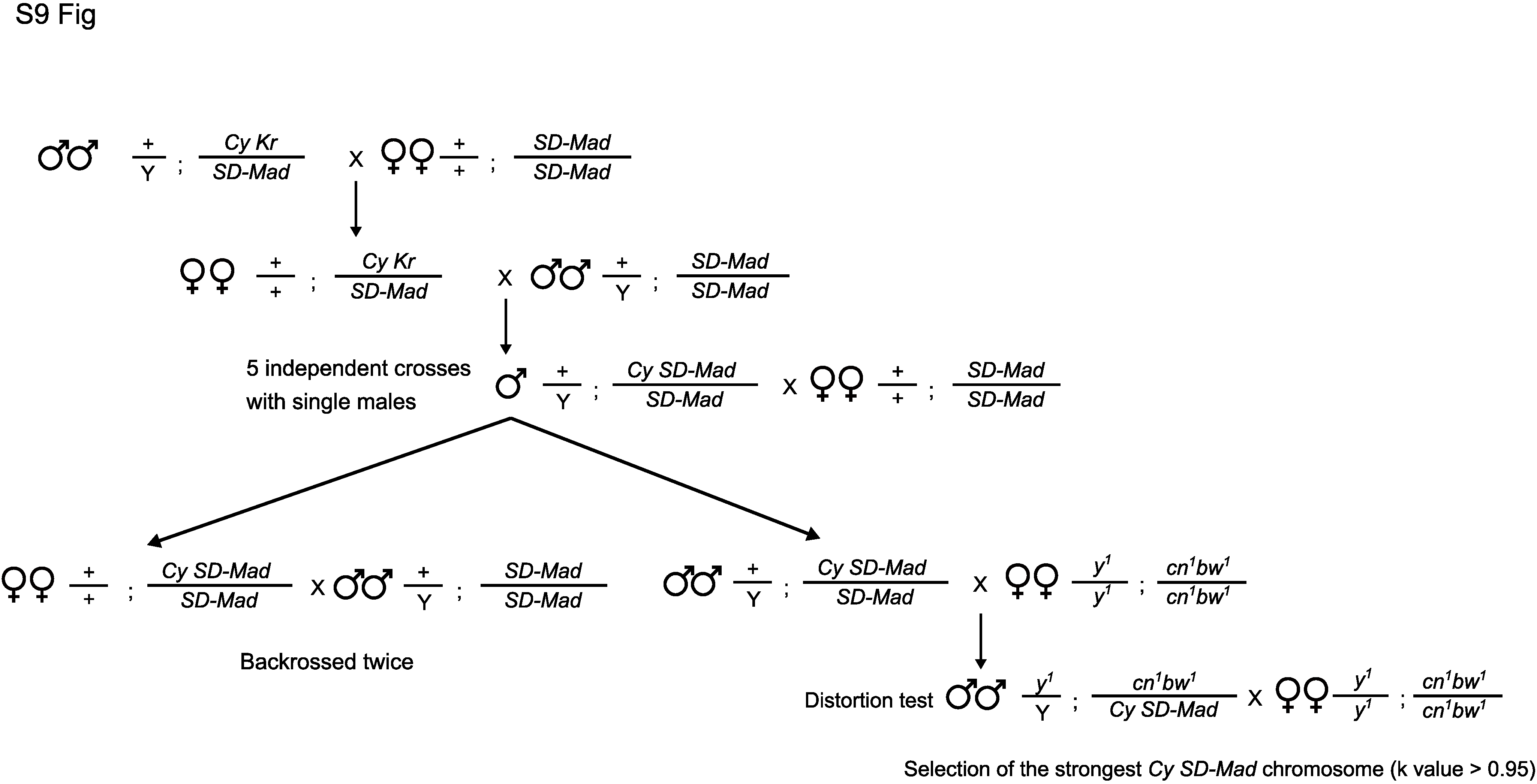

Supplement: S9 Fig — (TIF) [file pgen.1009662.s009.tif]
