# Supplementary material for: Distinct spermiogenic phenotypes underlie sperm elimination in the Segregation Distorter meiotic drive system
Source: PLoS Genet. 2021 Jul 6;17(7):e1009662. doi: 10.1371/journal.pgen.1009662 (PMC8284685; doi:10.1371/journal.pgen.1009662)
Supplement: Attachment — Submitted filename: 113576_1_rebuttal_2019160_qq2cpk.pdf [file pgen.1009662.s010.pdf]

We thank both reviewers for their comments on our manuscript. Our initial responses to their specific comments and plan to modify the manuscript are described bellow.

<u>Response to reviewer #1</u>

> Figure 1C is difficult to interpret. Am I supposed to see anything in particular in the two insets? Please provide a descriptive interpretation of the inset and let the reader know if anything in particular is to be noted.

We agree that it is a bit difficult to interpret although the goal of these images is to show that spermatogenesis appears globally not disturbed until the histone-to-protamine transition in distorter males. We will change the legend of the figure to clarify this particular point.

> Figure 1D. Elsewhere, in supplemental S1, they have an image for SD5/CyO. This should be provided here as a control. As presented, the genetics don't prove that the interaction between SD5 and Gla is the cause of the phenotype. As presented in the figure, the effect could be caused by SD5 alone, independent of Gla. In S1, this is not the case - SD5 with CyO doesn't produce the phenotype. Likewise, I think they should provide the SD5/CyO image in S1A in Figure 1C.

We can add the images of the *SD5/CyO* genotype (currently in FigS1) in Fig1C (whole testis) and in Fig1D (single cyst). We also suggest to present in this figure the other distorter genotype *cn bw/CyO* (which is currently in Fig S1). However, because the modified figure is going to be too big, we also suggest to split Figure 1 in two Figures with Figure 2 presenting FISH results including all controls. In this case, we will remove the supplemental figure 1.

> Figure 1E. These images are the formal proof (especially for Gla/SD5 genotype) that the large Rsp array is on the chromosomes that seem destined for removal from the cyst. However, there is no control. The authors should provide FISH results for the genotypes Gla/CyO and, ideally, also cn1 bw1/Cyo.

We agree with reviewer #1 and will provide images of the *Gla/CyO* control and *cn1 bw1/Cyo* in a new Figure 2 as explained above.

> Figure 2. Keeping consistent with other figures, can the Gla/SD5 panel be in the middle?

Yes. We swapped the Gla/SD5 and cn bw/SD-Mad panels.

Also, shouldn't there be SD5/CyO in Figures 2, 3 and 4, to demonstrate that the phenotypes are the result of the interaction rather than just SD5? I am OK with providing just the cn 1 bw1/SD-Mad here alone, since it is simply contrasted with Gla/SD5.

We agree that it would be better to show also the *SD5/CyO* controls. However, we chose to show only one control (*Gla/CyO*) to make the figures easier to read. We thus suggest to provide all images of the *SD5/CyO* genotype in supplemental figures.

> Figure 3A. In the scheme, can you provide greater detail as to where F-Actin is expected?

The scheme was modified to clarify this point.

> Figure 3B. It is stated that there is a size difference in the nuclei for IC stage and greater variation in ProtB-GFP staining within bundles. Can there be an effort to quantify these observations?

It would be difficult to quantify ProtB-GFP signal intensity and nuclear size in IC stage cyst because nuclei are very close to each other. The best way would be to squash testes to spread spermatid nuclei but there might be a bias on nuclear size/shape due to the squashing procedure. In addition, on squashed preparations, it is difficult to be sure that the nuclei analyzed and compared belong to the same cyst. We agree that quantification would help to describe the phenotype better but we think that the best read-out of the different *SD* phenotypes is the quantification of number of abnormally compacted nuclei in seminal vesicles which is provided later in the manuscript.

> Figure S4. There doesn't appear to be the same phenotype for Gla/SD-Mad (DAPI, ProtB-GFP) in post-IC stage bundles compared to what is seen in 3C for Gla/SD-5. In particular, in figure 3, the defective nuclei seem to be trailing, but in S4, while the bundle appears disorganized, there doesn't appear to be the trailing nuclei. Is this difference real or is it just the result of a single picture contrast? Some clarification could be helpful.

Actually, the images that were shown on Figure 3C for *Gla/SD5* post-IC probably show the *SD5* nuclei of one cyst (the normal one) and the *Rsp* nuclei being eliminated from another cyst (these are trailing behind nuclei which are too far to be included in the same image). We thus changed the images for *Gla/SD5* for an image which looks like the one shown for *Gla/SD-5* genotype for clarity.
We did not mention this observation in the manuscript but we actually see cysts in which abnormally-shaped nuclei are trailing behind the normal nuclei and sometimes IC cones around the abnormally shaped nuclei seem to be stuck close to the normal nuclei which are already individualized. It might be possible that IC progression around abnormal nuclei is slowed down compared to normal nuclei. The difference could not reflect different phenotypes but more likely different states of a dynamic process.

Response to reviewer #2

Reviewer #2 had no specific comments.
