# Supplementary material for: Distinct spermiogenic phenotypes underlie sperm elimination in the Segregation Distorter meiotic drive system
Source: PLoS Genet. 2021 Jul 6;17(7):e1009662. doi: 10.1371/journal.pgen.1009662 (PMC8284685; doi:10.1371/journal.pgen.1009662)
Supplement: Attachment — Submitted filename: ResponsetoReviewers-PLoS-Genet.docx [file pgen.1009662.s011.docx]

We thank all reviewers for their comments on our manuscript. The modifications and corrections that we made in the manuscript are described below together with our responses to specific Reviewer comments.

**Reviewer #1** did not raise any specific issue.

**Reviewer #2:**

*The authors found improperly condensed sperm nuclei from the cn1 bw1/Sd-Mad males in the seminal vesicle. On page 15, they stated: “Although we know that these sperm cannot give rise to viable progeny, the timing of their elimination remains to be determined.” It would be interesting, important, and potentially easy to examine some embryos fertilized by strong SD males to see if these sperm are capable of penetrating the egg and if so, what happens afterward.*

> We agree with Reviewer#2. It would indeed be interesting to know if the abnormally compacted sperm nuclei observed in seminal vesicles of some SD male genotypes are actually transferred to female and fertilize eggs. A direct strategy would have been to perform DNA-FISH on embryos but these experiments are technically challenging and difficult to quantify. To indirectly address this question, we have instead stained sperm storage organs (seminal receptacles) in females mated to *cn^1^bw^1^/ SD-Mad* males with the anti-dsDNA antibody to detect abnormally compacted sperm nuclei. Over 10 females analyzed, we only observed ten anti-dsDNA positive nuclei in the seminal receptacle of one female. These observations suggest that a large majority of abnormally compacted *Rsp* sperm nuclei from *cn^1^bw^1^/ SD-Mad* males are lost before reaching the sperm storage organs. These results are now presented in a supplemental figure (S4-Fig).

We added the following sentences in the results (pp 11-12) " Importantly, since the *cn^1^ bw^1^*/*SD-Mad* males exhibited very high levels of segregation distortion (*k* value 0.998; Table 1), we inferred that these abnormally condensed sperm cells were unable to fertilize eggs. In fact, abnormally compacted sperm nuclei were only very rarely observed in sperm storage organs of females mated to *cn^1^ bw^1^*/*SD-Mad* males (S4 Fig)."

*In the discussion, the authors state: “The checkpoint may function to selectively remove abnormal spermatids, thus avoiding the production of defective spermatozoa that could impact progeny survival.” It might be better to state that the checkpoint effect could mitigate a fecundity loss suffered by the male parent. This may be especially true if the under-condensed sperm are capable of undergoing fertilization but result in embryonic death.*

> We replaced the sentence by "The checkpoint may function to selectively remove abnormal spermatids to mitigate a fecundity loss suffered by the male parent."

*How does the checkpoint work to eliminate any defective sperm if all sperm in a bundle are interconnected by a common cellular cytoplasm? It seems difficult to imagine a checkpoint that operates to eliminate individual sperm within a bundle of otherwise normally developing sperm. Is there evidence of checkpoints acting on certain individual nuclei and not others, all within a syncytium? Further elaboration on this matter would be helpful.*

*>* There are actually examples of selective elimination of nuclei sharing a same cytoplasm: at fertilization in *Arabidobsis thaliana* for instance (Maruyama et al. 2015) or the syncytiotrophoblast in the mammalian placenta (Huppertz et al. 1998). In the *Drosophila* embryo which starts to develop as a syncytium, damaged nuclei are eliminated through a mechanism called nuclear fallout. This occurs during the blastoderm formation when nuclei migrate at the periphery and involves a Chk2-dependent nuclear retention of specific mRNAs necessary for the integration and maintenance of nuclei (Iampetro et al. 2014). During *Drosophila* spermatogenesis, it has been observed by us and others (Tokuyasu *et al.,* 1972) that some spermatid nuclei within a same cyst do not individualize suggesting the implication of a checkpoint. Although the underlying mechanism is totally unknown in this case, it does seem plausible that such mechanism could operate within a spermatid cyst. To clarify this point in the discussion we added the following sentences: " This implies that a molecular mechanism discriminates abnormal nuclei within the cyst cytoplasm. In the *Drosophila* embryo, which starts to develop as a syncytium, damaged nuclei are eliminated during the blastoderm formation when nuclei migrate to the periphery through a Chk2-dependent checkpoint (Iampetro et al. 2014). A similar mechanism might thus operate during spermiogenesis to selectively remove abnormal spermatids. "

*The authors mention the possibility that piRNA perturbation may either directly or indirectly cause defective Rsp heterochromatin formation. Why would such an effect on heterochromatin formation be specific to the Rsp satDNA ? There are many other abundant satDNAs in the fruit fly genome that also presumably utilize the trans-nuclear membrane transport system and, thus, should also be similarly affected under this model.*

> The fact that a mutant in the argonaute protein genes (*aubergine, piwi*) behaves as an enhancer of SD supports a role of piRNAs in the system (Gell et al. 2013). Furthermore, the *Rsp* satDNA, as other complex satDNAs, is transcribed into long non coding RNA in the female and male germline and processed into piRNAs (Wei et al. 2020 bioRxiv). However, we agree that the specific implication of *Rsp* in SD remains an unsolved mystery (“The simple fact is that we still lack a cogent model for how Sd and Rsp interact to cause distortion” Larracuente and Presgraves 2012). It might be the interaction between Sd-RanGAP and other uncharacterized genetic elements required to induce -together with Sd- high levels of distortion. Another hypothesis is that *Rsp* may have a specific function during spermatogenesis that the other satDNAs do not have. We added a sentence to underline this paradox in the discussion (p.17) : " In any case, what makes the specificity of *Rsp* in the SD system relative to other large satDNAs is still an unsolved mystery. "

Minor comments

*Top of page 5 – “compaction is primary driven” should be “primarily driven”*

> It has been corrected

*Title on top of page 12 should read “…when Rsp carries…”*

> We modified the title accordingly.

*Toward the top of page 12, “…with single-molecule sequencing long reads and…” I think should read “…with single-molecule, long read sequencing and…”*

> We replaced by "single-molecule long-read sequencing" (without comma)

*Top of page 14, “…levels were lower…” Lower in comparison to what? It would help to be more specific.*

> We replaced the sentence by "....we noticed that distortion levels were lower when we used females, instead of males, from the *RAL-313*, *RAL-309* and *RAL-380* strains in our crosses to produce *SD*/*SD^+^* males..."

*Toward the top of page 14, “…stained their testes with DAPI and F-actin…” One cannot stain with F-actin, but F-actin is stained with certain reagents.*

> We agree with Reviewer#2 and replaced by “…stained their testes with DAPI and phalloidin…” in this sentence and in other places in the manuscript.

**Reviewer #3**

*Previous Rev 1 had some good points but it doesn’t appear that all suggestions were implemented by the authors. For example, the authors say they were going to modify Fig 2 to include FISH data on control genotypes, but this doesn’t appear to have been done.*

> We actually did not make all the changes asked by Review Commons reviewer 1 when we submitted to PLoS Genetics. In the mail we received from Review Commons, it was written "At this stage, a full revision is NOT expected! " and we were asked to "submit [a] response/initial revision plan". We thus submitted our article to PLoS Genetics together with our revision plan. These modifications are now incorporated in this revised version. In particular, we made two figures (Fig1 and Fig2) with former Fig1. The new Fig1 now shows whole testes in the four genotypes *Gla/CyO, SD5/CyO, Gla/SD5* and *cn^1^ bw^1^/ SD-Mad* and Fig2 shows images of spermatid cysts in IF (with DAPI and ProtB-GFP) and the corresponding FISH data. The magnification and clarity of the insets in Fig 1 have been improved (Histone and Vasa channels have been removed and arrowheads have been added).

We also made a Supplementary Figure 2 (S2 Fig) presenting the images of *SD5/CyO* controls for Figure 3, 4 and 5 and swapped *Gla/SD5* and *cn bw/SD-Mad* panels in Fig 3 (former Fig 2) to be consistent with the order in the other figures as suggested by Review Commons' reviewer 1.

*In Fig 1, it looks like more than a few nuclei staining with Rsp are normal in shape.*

> We modified the legend of this figure (now Fig 2). We wrote "In *cn^1^ bw^1^/SD-Mad*, both abnormally-shaped and normally-shaped (arrowhead) spermatid nuclei carry a second chromosome with a large *Rsp* satDNA block." We also modified the sentence in the text on p.7 for "Moreover, the DNA-FISH *Rsp* probe stained both normally and abnormally-shaped spermatid nuclei (Fig 2B)."

Minor points.

*p.6 ‘classical’ is unnecessary, what would a non-classical chromosome be?*

> We removed the term classical.

*p.7 should be ‘and a few nuclei with an apparent …’*

*>* We modified the sentence as "Moreover, the DNA-FISH *Rsp* probe stained both normally and abnormally-shaped spermatid nuclei (Fig 2B)." to take into account the comment of reviewer 3 ("*In Fig 1, it looks like more than a few nuclei staining with Rsp are normal in shape.").*

*p. 9 stained-somatic should be stained somatic
p. 12 section title at top. Should be Rsp carries 2000…*

*p. 16 and then lead to …
Fig 1 legend. Spermatids become individualized and coiled …; appear abnormally shaped and are eliminated ...
Fig 3 legend. Is variable from one cyst to another …
Fig 8 legend. “too important” should be “too severe” or similar.*

> All typos mentioned above were corrected.

*Fig 2 legend. Cn bw / Sd-Mad should be described before Gla/SD5 in order to match left-to-right order of the figure.*

> We did not modify the legend but instead Fig 3 (former Fig2) as explained above.

*Also, what does the purple coloring indicate in the two SD genotypes.*

>The purple coloring indicates chromatin compaction defects in the whole nucleus due to perturbation of protamine incorporation. We agree with Reviewer 3 that the color code is quite not clear on the scheme. We thus modified the figure as follows: purple dots represent a modified/perturbed chromatin state and this color code has been added in the *Rsp* satDNA of SD male spermatid nuclei. Then purple dots were added in the whole nucleus after the incorporation of protamine to represent chromatin compaction defects.

**Summary of figure modifications**

Fig1 is former Fig1A-C with additional control genotypes in Fig 1C

Fig2 is former Fig 1D-E with additional control genotypes

Fig3 is former Fig2

Fig4 is former Fig3

Fig5 is former Fig4

Fig6 is former Fig5

Fig7 is former Fig6

Fig8 is former Fig7

Fig9 is former Fig8

S1 Fig shows the SD5/CyO controls of Figs 2-4

S2 Fig did not change

S3 Fig did not change

S4 Fig is a new S Fig showing female seminal receptacle

S5 Fig is former S4 Fig

S6 Fig is former S5

S7 Fig did not change

S8 Fig is former S6 to follow the citation order of S7 and S8 in the text.

S9 Fig is former S8 Fig
